# Suppression of Estrogen Receptor Transcriptional Activity by Connective Tissue Growth Factor

**DOI:** 10.1371/journal.pone.0020028

**Published:** 2011-05-24

**Authors:** Long Cheng, Zhihong Yang, Xiaohui Wang, Yuanyuan Jiao, Xiangyang Xie, Jing Lin, Hao Zhang, Juqiang Han, Kai Jiang, Qinong Ye

**Affiliations:** 1 Department of Medical Molecular Biology, Beijing Institute of Biotechnology, Beijing, People's Republic of China; 2 Department of Clinical Laboratory, First Affiliated Hospital, Chinese PLA General Hospital, Beijing, People's Republic of China; University Paris Diderot-Paris 7, France

## Abstract

Secreted growth factors have been shown to stimulate the transcriptional activity of estrogen receptors (ER) that are responsible for many biological processes. However, whether these growth factors physically interact with ER remains unclear. Here, we show for the first time that connective tissue growth factor (CTGF) physically and functionally associates with ER. CTGF interacted with ER both in vitro and in vivo. CTGF interacted with ER DNA-binding domain. ER interaction region in CTGF was mapped to the thrombospondin type I repeat, a cell attachment motif. Overexpression of CTGF inhibited ER transcriptional activity as well as the expression of estrogen-responsive genes, including pS2 and cathepsin D. Reduction of endogenous CTGF with CTGF small interfering RNA enhanced ER transcriptional activity. The interaction between CTGF and ER is required for the repression of estrogen-responsive transcription by CTGF. Moreover, CTGF reduced ER protein expression, whereas the CTGF mutant that did not repress ER transcriptional activity also did not alter ER protein levels. The results suggested the transcriptional regulation of estrogen signaling through interaction between CTGF and ER, and thus may provide a novel mechanism by which cross-talk between secreted growth factor and ER signaling pathways occurs.

## Introduction

Estrogen receptors (ERα and ERβ), hormone-dependent transcription factors belonging to the steroid/thyroid-hormone-receptor superfamily, play important roles in the development and progression of steroid hormone-dependent cancers, including breast cancer, ovarian cancer and cervical cancer [1], [2]. ERs share structural similarity characterized by several functional domains. N-terminal estrogen-independent and C-terminal estrogen-dependent activation function domains (AF1 and AF2, respectively) contribute to the transcriptional activity of the two receptors. The DNA binding domain (DBD) of the ERs is centrally located. The ligand binding domain, overlapping AF2, shows 58% homology between ERα and ERβ. The DBD is identical between the two receptors except for three amino acids. However, the AF1 domain of ERβ has only 28% homology with that of ERα ERα and ERβ have similar binding affinities for estrogen and their cognate DNA binding site, which is probably due to the high degree of sequence homology they share in their ligand and DNA binding domains.

Traditionally, ERs are thought to be intracellular transcription factors that bind to the promoters of the estrogen-responsive target genes, such as pS2 and cathepsin D [3]. Recently, estrogen was shown to mediate rapid non-genomic pathyways through interaction with membrane receptors, especially membrane ERs [4], [5]. Membrane ERs also play an important role in indirect regulation of ER transcriptional activity. Membrane ERα-mediated non-genomic estrogen actions require a large protein complex, comprising ERα, the adaptor protein Shc and insulin-like growth factor 1 receptor (IGF-1R).

Estrogens, acting via ER, are important regulators of the growth and differentiation of many estrogen-regulated tissues, including ovary, uterus, mammary gland, and brain. Secreted growth factors, such as epidermal growth factor (EGF) and insulin-like growth factor-1 (IGF-1), also mimic estrogens in their ability to increase ER transcriptional activity as well as the expression of ER target genes [6], [7]. EGF and IGF-1 exerts some of their biological responses in an ER-dependent manner, suggesting the cross-talk of growth factors with ER signaling pathway. However, whether these growth factors physically interact with ER remains unclear. In this study, we have identified and characterized a novel ER-interacting protein, connective tissue growth factor (CTGF). CTGF is a secreted protein that belongs to the CCN family, including Cyr61 (cysteine-rich protein 61), CTGF, Nov (nephroblastoma overexpressed), WISP-1 (Wnt-1-induced secreted protein 1), WISP-2, and WISP-3 [8]–[10]. CTGF consists of four domains from the N-terminus to the C-terminus: the insulin-like growth factor binding protein domain (IGFBP), the Von Willebrand factor type C repeat (VWC), the thrombospondin type I repeat (TSP-1) and the C-terminal domain (CT). The biological properties of CTGF involve cell adhesion, migration, proliferation, survival, differentiation and tumorigenesis [11]. Here, we show that CTGF physically interacts with ERα and ERβ, and functionally inhibits ER-mediated estrogen signaling.

## Materials and Methods

### Plasmids

The reporter constructs ERE-Luc [12], pS2-Luc [12], ARE-Luc [13] and pFC31-Luc [14], eukaryotic expression vectors for ERα and ERβ [15], prokaryotic expression vectors for glutathione S-transferase (GST)-tagged ERα, ERα(180–282) and ERβ [13]–[15], and the yeast expression vectors pAS2-ERβ(1–167), pAS2-ERβ(131–324) and pAS2-ERβ(286–530) [15] have been described previously. The yeast expression vectors pAS2-ERα(1–185), pAS2-ERα(180–282) and pAS2-ERα(282–595) were generated by inserting the corresponding cDNA fragments into pAS2-1 (Clontech). FLAG-tagged CTGF and its mutants were created by cloning the corresponding sequences into a pcDNA3 vector (Invitrogen) linked with FLAG at the carboxyl terminus. Plasmids encoding GST-CTGF and its mutants were prepared by cloning the corresponding sequences into pGEX-KG (Amersham Pharmacia Biotech). His-tagged CTGF and Nov were generated by inserting the corresponding cDNAs into pET28a (Novagen).

### Yeast two-hybrid assay

The bait plasmid pAS2-ERβ(131–324) and a human mammary cDNA prey library (Clontech) were sequentially transformed into Saccharomyces cerevisiae strain CG1945 according to the manufacturer's protocol (Clontech). Transformants were grown on synthetic medium lacking tryptophan, leucine and histidine but containing 1 mM 3-aminotriazole. The candidate clones were rescued from the yeast cells and re-transformed back to the same yeast strain to verify the interaction between the candidates and the bait. The unrelated bait plasmid pAS2-lamin C was used as a negative control.

### GST pull-down assay

The GST- and His-fusion proteins were expressed and purified according to the manufacturers' protocols (Amersham Pharmacia and Qiagen). The purified GST fusion protein bound to glutathione-Sepharose beads were incubated with ^35^S-labeled in vitro translation products or purified His-fusion proteins, and the adsorbed proteins were analyzed as previously described [16].

### Coimmunoprecipitation

Cell lysates were prepared in lysis buffer (50 mM Tris at pH 8.0, 500 mM NaCl, 0.5% Nonidet P-40, 1 mM dithiothreitol, and protease inhibitors) and mixed with conditioned media. The mixture was subjected to immunoprecipitation with anti-FLAG agarose beads (Sigma-Aldrich) as previously described [16]. Immunoblot analysis was performed with anti-ERα (Santa Cruz Biotech) or anti-ERβ (Abcam).

### Enzyme-linked immunosorbent assay (ELISA) for protein-protein binding

The 96-well plates were coated with mouse anti-human CTGF monoclonal antibody (Santa Cruz Biotech) diluted in 100 mM carbonate buffer at pH 9.6 (1∶1000 v/v) overnight at 4°C. The wells were washed with PBST (0.05% Tween-20, PBS pH 7.5) and blocked with PBSTM (0.05% Tween-20, 5% dried milk, PBS pH 7.5) for 1 h at room temperature. Cell lysates together with conditioned media were then incubated in wells for 2 h. After washes with PBST, rabbit anti-human ERα antibody (Sigma-Aldrich) (1∶2500 v/v) or normal rabbit serum (Santa Cruz Biotech) diluted in PBSTM was incubated in wells for 1 h. Following washes with PBST, the wells were incubated with HRP-conjugated goat anti-rabbit IgG (1∶2500 v/v) (Santa Cruz Biotech). After final washes with PBS, 50 µl of TMB reagent (Sigma-Aldrich) was added. After 30 minutes the reaction was stopped with 50 µl 1 M H_2_SO_4_. Absorbance at 415 nm was measured with a plate reader.

### Immunofluorescence assay

Cells on glass coverslips were fixed with 1.6% paraformaldehyde for 30 minutes, permeabilized with 0.2% Triton X-100 for 5 minutes, and blocked in 1% normal goat serum for 1 hour. The coverslips were then incubated with rabbit anti-ERα (Sigma-Aldrich) or mouse anti-CTGF (Santa Cruz Biotech), followed by incubation with goat anti-rabbit IgG (Santa Cruz Biotech) or goat anti-mouse IgG (Santa Cruz Biotech) secondary antibodies. Nuclei were counterstained with 0.2 µg/ml DAPI. Confocal images were collected using a Radiance2100 confocal microscope (Bio-Rad).

### Luciferase reporter assay

MCF7, ZR75-1 and Hela cells were routinely grown in DMEM (Invitrogen) supplemented with 10% fetal bovine serum (FBS). For transfection, cells were seeded in 12- or 24-well plates containing phenol red-free DMEM medium supplemented with 10% charcoal-stripped FBS (Hyclone). The cells were transfected using Lipofectamine 2000 (Invitrogen) with 0.1 or 0.2 µg of the luciferase reporter ERE-Luc or pS2-LUC plus various amounts of expression vector for CTGF or recombinant human CTGF, EGF or IGF-1 proteins (ProSpec), with or without 25 ng or 50 ng of ERα or ERβ expression vector. β-galactosidase reporter was used as an internal control. After treatment with 10 nM of 17β-estradiol (E2) for 24 h, the transfected cells were collected. Luciferase activity was assessed as described [17].

### RNA interference

The target sequences for differential knockdown of CTGF protein expression are GAAGAACATGATGTTCATC (siRNA1) and GTACCAGTGCACGTGCCTG (siRNA2), respectively. The target sequences were cloned into pSilencer2.1-U6neo according to the manufacturer's protocol (Ambion). Plasmid pSilencer2.1-U6neo negative control was used as a negative control vector. Transfection of the plasmid-based siRNAs into mammalian cells was carried out using Lipofectamine 2000 (Invitrogen). Knockdown of CTGF protein was confirmed by Western blotting with anti-CTGF (Santa Cruz Biotech).

### Stable transfection of CTGF

MCF7 cells were transfected with FLAG-tagged CTGF or empty vector using Lipofectamine 2000 (Invitrogen), and the transfected MCF7 cells were selected in 500 µg/ml G418 (Invitrogen) for approximately 2 months. Pooled clones or individual clones were screened by immunoblot with anti-FLAG (Sigma-Aldrich). Similar results were obtained with individual clones or pooled clones.

### Electrophoretic mobility shift assay (EMSA)

The ERE (5′-AGCTCTTTGATCAGGTCACTGTGACCTGACTTT-3′) or mutant ERE (EREM; 5′-AGCTCTTTGATCAGTACACTGTGACCTGACTTT-3′) probes were labeled with Biotin 3′-End DNA Labeling kit (Pierce) as instructed by the manufacturer. EMSA was performed using LightShift Chemiluminescent EMSA kits (Pierce). Briefly, binding reactions containing 10 µg of nuclear extracts and 1 nmol of oligonucleotide were performed for 30 min in binding buffer (2.5% glycerol, 0.05% Nonidet P-40, 50 mM KCl, 5 mM MgCl2, 1 mM EDTA, 10 mM Tris, pH 7.6, and 50 ng of poly(dI-dC)). Protein-nucleic acid complexes were resolved using a nondenaturating polyacrylamide gel consisting of 6% acrylamide, and transferred to a nylon membrane. The membrane was incubated in blocking solution followed by incubation with streptavidin-peroxidase. After extensive washing, signal was detected with chemiluminescence solution.

### Real-time RT-PCR

Total RNA was isolated using TRIzol Reagent (Invitrogen) and reverse transcribed using SuperScript II Reverse Transcriptase (Invitrogen). Real-time PCR was performed with ERα-, CTGF-, GAPDH-, and β-actin-specific primers. The sense primer for ERα was 5′-CCACCAACCAGTGCACCATT-3′ and the antisense primer was 5′-GGTCTTTTCGTATCCCACCTTTC-3′. For CTGF, the sense primer was 5′-GCAGGCTAGAGAAGCAGAGC-3′ and the antisense primer was 5′-ATGTCTTCATGCTGGTGCAG-3′. The sense primer for GAPDH was 5′-ACCACAGTCCATGCCATCAC-3′ and the antisense primer was 5′-TCCACCACCCTGTTGCTGTA-3′. For β-actin, the sense primer was 5′-ATCACCATTGGCAATGAGCG-3′ and the antisense primer was 5′-TTGAAGGTAGTTTCGTGGAT-3′. The fold change in expression of ERα or CTGF was calculated using the 2^−ΔΔCt^ method, with GAPDH or β-actin as an internal control.

### Western blot

Approximately 50 µg of protein samples were separated by SDS-polyacrylamide gel electrophoresis and blotted to a nitrocellulose membrane. Blotted membranes were blocked overnight at 4°C in TBST containing 5% nonfat milk. Blots were incubated with primary antibodies diluted in TBST containing 5% nonfat milk for 1 h at room temperature. After washing extensively with TBST, membranes were incubated with the appropriate horse radish peroxidase-conjugated secondary antibody (Santa Cruz Biotech), followed by chemiluminescent detection according to the manufacturer's instructions (Pierce). The primary antibodies used in this study are as follows: mouse anti-FLAG (Sigma-Aldrich), mouse anti-His (GE Healthcare), rabbit anti-ERα (Santa Cruz Biotech), rabbit anti-ERβ (Abcam), mouse anti-CTGF (Santa Cruz Biotech) and rabbit anti-GAPDH (Santa Cruz Biotech).

### Statistical analysis

The values are expressed as means ± SD. Statistical significance in the luciferase activity experiments between two constructs was assessed by Student's *t*-test. When doses and increasing concentrations were compared, statistical significance was determined by one-way analysis of variance (ANOVA). A *P* value<0.05 was considered statistically significant.

## Results

### Interaction of CTGF with ERα and ERβ in yeast cells

**Figure 1:**
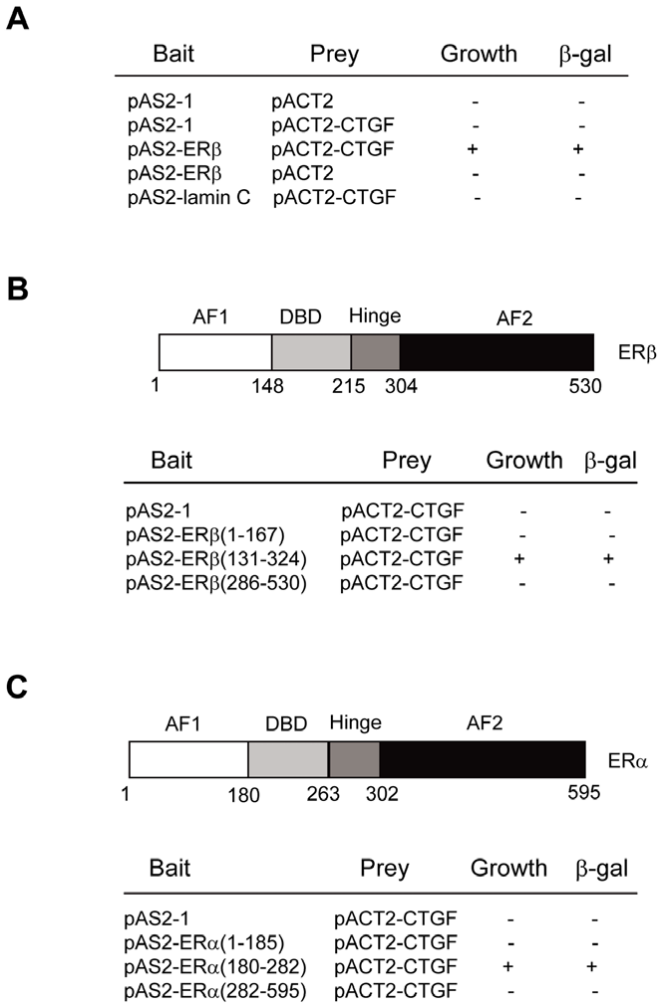
Identification and characterization of the CTGF-ER interaction in yeast cells. (A) Identification of CTGF as an ERβ-interacting protein by the yeast two-hybrid system. Yeast CG1945 cells were transformed with the indicated plasmids (bait and prey) and grown on SD/-Trp-Leu and SD/-Trp-Leu-His. Colonies grown on SD/-Trp-Leu or SD/-Trp-Leu-His were tested for β-galactosidase activity (LacZ). Positive interaction is indicative of His- (growth) and LacZ- (β-gal) positive colonies. (B) Mapping of the CTGF interaction region in ERβ. CG1945 cells were transformed with the indicated constructs and analyzed as in (A). Schematic diagram of the ERβ protein is shown at the top. (C) Mapping of the CTGF interaction region in ERα. CG1945 cells were transformed with the indicated plasmids and analyzed as in (A). Schematic diagram of the ERα protein is shown at the top.

To identify proteins that could be involved in regulation of estrogen signaling, we screened a human mammary cDNA library using amino acids 131–324 containing the ERβ DBD and hinge domains as bait in the yeast two-hybrid system. CTGF was identified as an ERβ-interacting protein. As shown in Fig. 1A, transformation of yeast cells with CTGF and ERβ(131–324), but not with other control plasmids, activate the his (growth) and lacZ (β-gal) reporter genes. CTGF did not interact with ERβ(1–167) containing the AF1 and ERβ(286–530) containing the AF2 (Fig. 1B), suggesting the specific interaction of CTGF with ERβ(131–324).

Since CTGF specifically interacts with ERβ DBD and hinge domains, and the DBD of ERβ has 96% homology with that of ERα, the possibility that the DBD of ERα may bind to CTGF was determined by yeast two-hybrid experiments. As shown in Fig. 1C, the ERα(180–282) containing the DBD specifically interacted with CTGF, but the ERα(1–185) containing the AF1 and the ERα(282–595) containing the hinge and AF2 regions did not. Taken together, these data suggest that the ER DBD domain is sufficient for CTGF binding in yeast cells.

### Interaction of CTGF with ERα and ERβ in mammalian cells and in vitro

**Figure 2:**
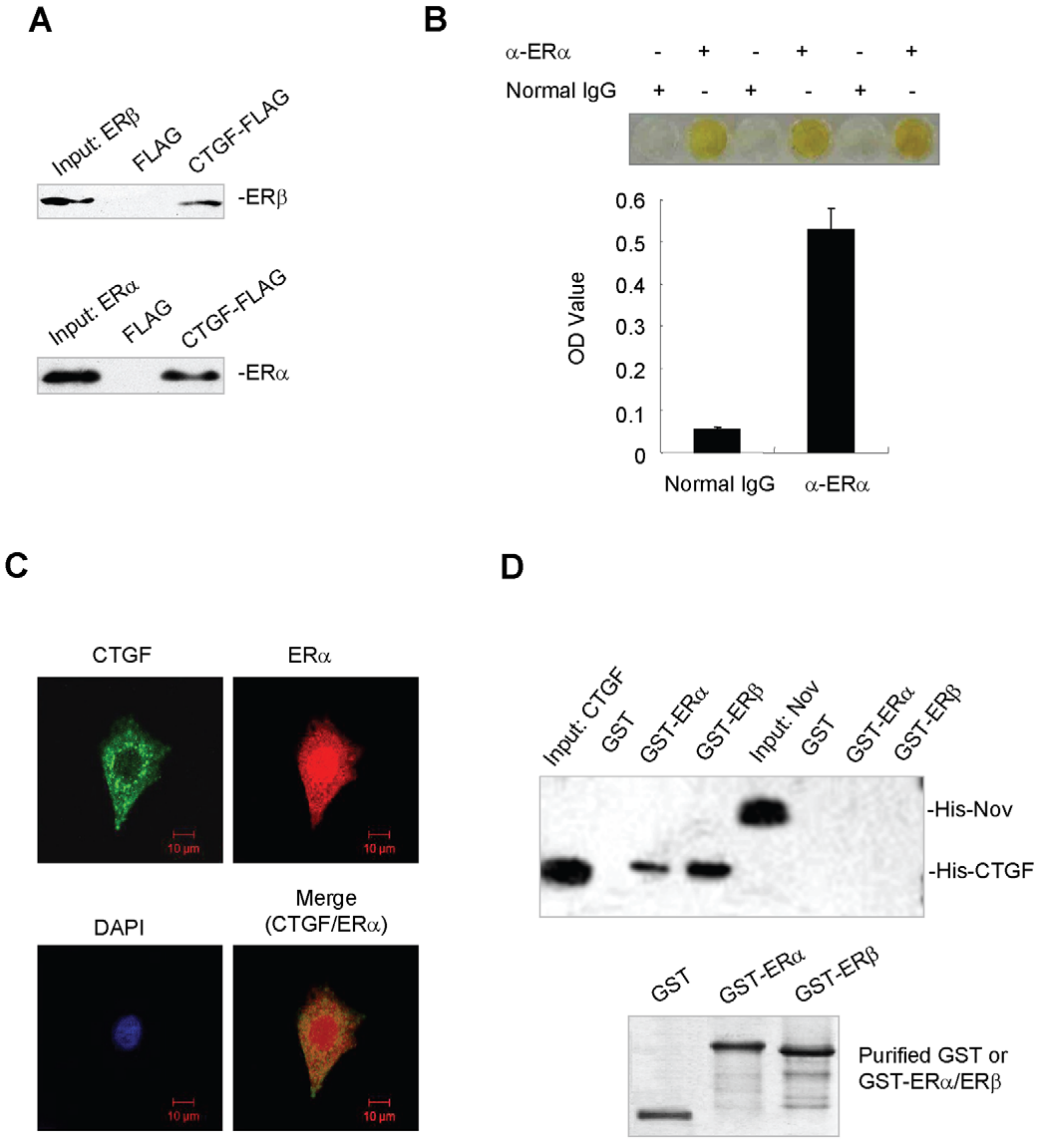
CTGF interacts with ERα and ERβ in mammalian cells and in vitro. (A) Interaction of CTGF with ERα and ERβ in mammalian cells. MCF7 cells were transfected with expression vector for FLAG-tagged CTGF (CTGF-FLAG) or empty (FLAG) plasmid in the presence of 17β-estradiol (E2). Conditioned medium from the FLAG- or CTGF-FLAG-transfected cells was incubated with MCF7 cell lysates. Immunoprecipitation (IP) was performed using anti-FLAG monoclonal antibody, and immunoblotted (IB) with anti-ERβ or anti-ERα. (B) Physiological interaction of CTGF with ERα by ELISA. CTGF monoclonal antibody-coated wells were incubated with MCF7 cell lysates together with conditioned media, followed by incubation with rabbit anti-human ERα antibody or normal rabbit serum. Absorbance at 415 nm (OD value) was measured with a plate reader. (C) Colocalization of endogenous CTGF with ERα. MCF7 cells were treated with 10 nm E2, immunostained for CTGF (green) and ERα (red), and counterstained for DNA with DAPI (blue). The images were captured by confocal immunofluorescence microscopy; original magnification, ×100. (D) Direct interaction of CTGF with ERα and ERβ. Glutathione–Sepharose beads bound with GST-ERα/ERβ or with GST were incubated with purified His-tagged CTGF or Nov. After washing the beads, the bound proteins were subjected to SDS–PAGE and Western blot with anti-His antibody.

To further confirm the interaction between CTGF and ERα/ERβ, coimmunoprecipitation experiments were performed with MCF7 breast cancer cells. FLAG-tagged CTGF coimmunoprecipitated ERα and ERβ (Fig. 2A). Since CTGF is expressed at relatively low level in ERα-positive cell lines, such as MCF7 (approximately 15 ng/10^7^ cells/24 h determined by ELISA) and ZR75-1 (approximately 12 ng/10^7^ cells/24 h) breast cancer cell lines (data not shown), a sensitive ELISA-based protein-protein binding detection method was employed to determine interaction of endogenous CTGF with endogenous ERα. As shown in Fig. 2B, endogenous CTGF specifically interacted with endogenous ERα in MCF7 cells. Moreover, immunofluorescence analysis of MCF7 cells showed that endogenous CTGF protein colocalized with endogenous ERα protein in both the cytoplasm and the membrane (Fig. 2C). The specificity of mouse anti-CTGF was confirmed by pre-incubation of the primary antibody with His-tagged CTGF protein or His control (Fig. S1A). Detection of CTGF was completely blocked by pre-incubating anti-CTGF with His-CTGF fusion protein but not by pre-incubating with His control. Furthermore, the staining pattern of endogenous CTGF in MCF7 cells was similar to that of FLAG-tagged CTGF fusion protein (Fig. S1B).

To determine whether CTGF directly interacts with ERα and ERβ in vitro, GST pull-down experiments were performed in which purified GST-ERα or GST-ERβ was incubated with purified His-CTGF or His-Nov. As shown in Fig. 2D, CTGF, but not Nov, another CCN family member, directly interacted with ERα and ERβ.

### Mapping of the ER and CTGF interaction regions

**Figure 3:**
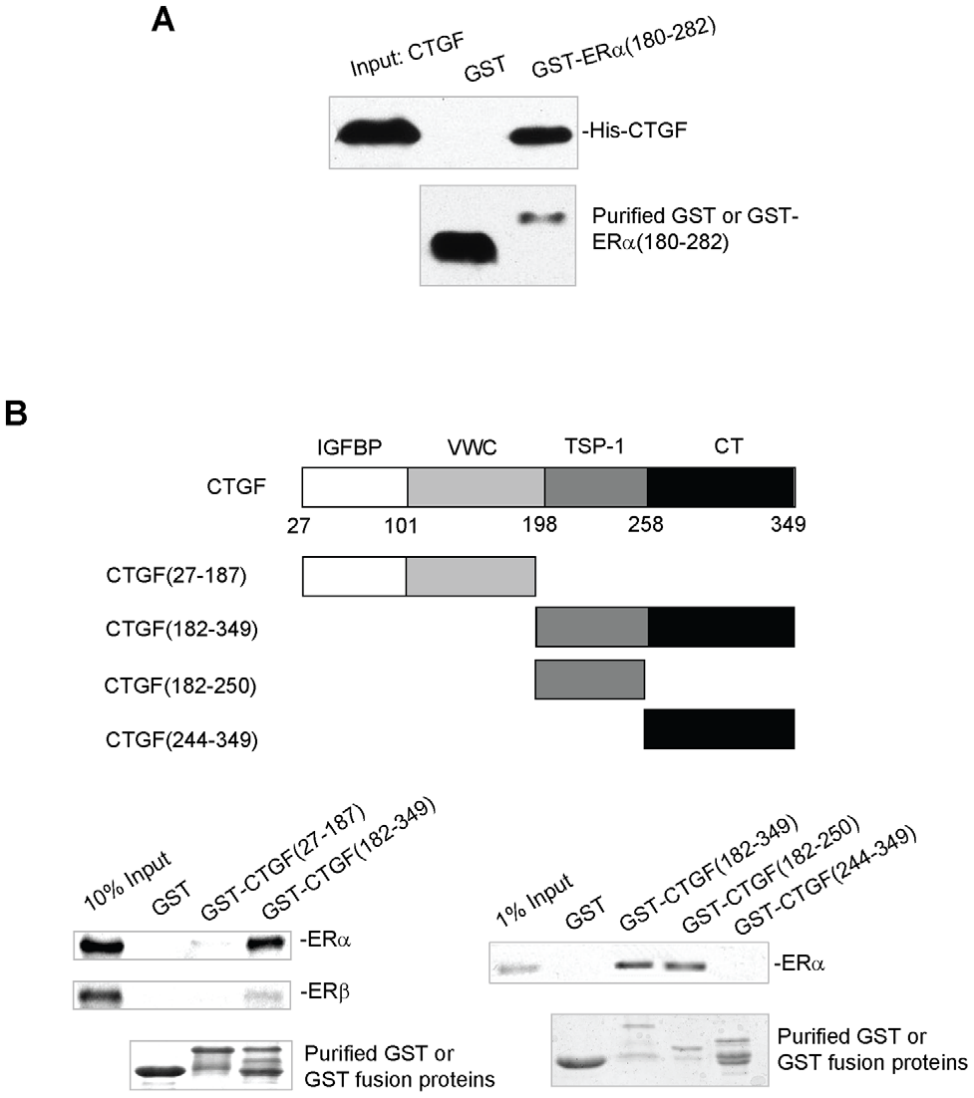
Mapping of the ERα and CTGF interaction domains. (A) Direct interaction of ERα DBD with CTGF. GST or GST-ERα(180–282) was incubated with purified His-CTGF. Bound proteins were analyzed as described in the legend to Figure 2D. (B) Mapping of the ER interaction region in CTGF. ^35^S-labelled in vitro translated ER was incubated with GST-CTGF(27–187), GST-CTGF(182–349), GST-CTGF(182–250) or GST-CTGF(244–349), or with GST. The bound proteins were subjected to SDS-PAGE followed by autoradiography. Also shown are schematic diagrams of the constructs used in this study.

ERα DBD was shown to interact specifically with CTGF in the yeast two-hybrid system (Fig. 1C). To further confirm the region of ERα required for its interaction with CTGF, GST pull-down experiments were performed in which GST-ERα(180–282) containing the DBD, and GST were incubated with purified His-tagged CTGF. Consistent with the results of the yeast two-hybrid, the direct interaction of CTGF with ERα DBD was also observed in the GST pull-down assay (Fig. 3A).

To define which domain of CTGF interacts with ER, GST pull-down experiments were performed again. The CTGF(182–349) fragment containing the TSP-1 and CT domains bound specifically to ERα and ERβ, whereas the CTGF(27–187) fragment containing the IGFBP and VWC domains but lacking the signal peptide did not bind ERα and ERβ (Fig. 3B, left panel). Further deletion analysis showed that the CTGF(182–250) containing the TSP-1 domain, but not the CTGF(244–349) containing the CT domain, is sufficient for ER binding (Fig. 3B, right panel). Compared with the results in Fig. 2, the CTGF(182–349) fragment interacted with ERβ very weakly. This might be due to different fusion proteins used and conformational changes in the fusion proteins.

### Overexpression of CTGF inhibits the transcriptional activity of ERα and ERβ

**Figure 4:**
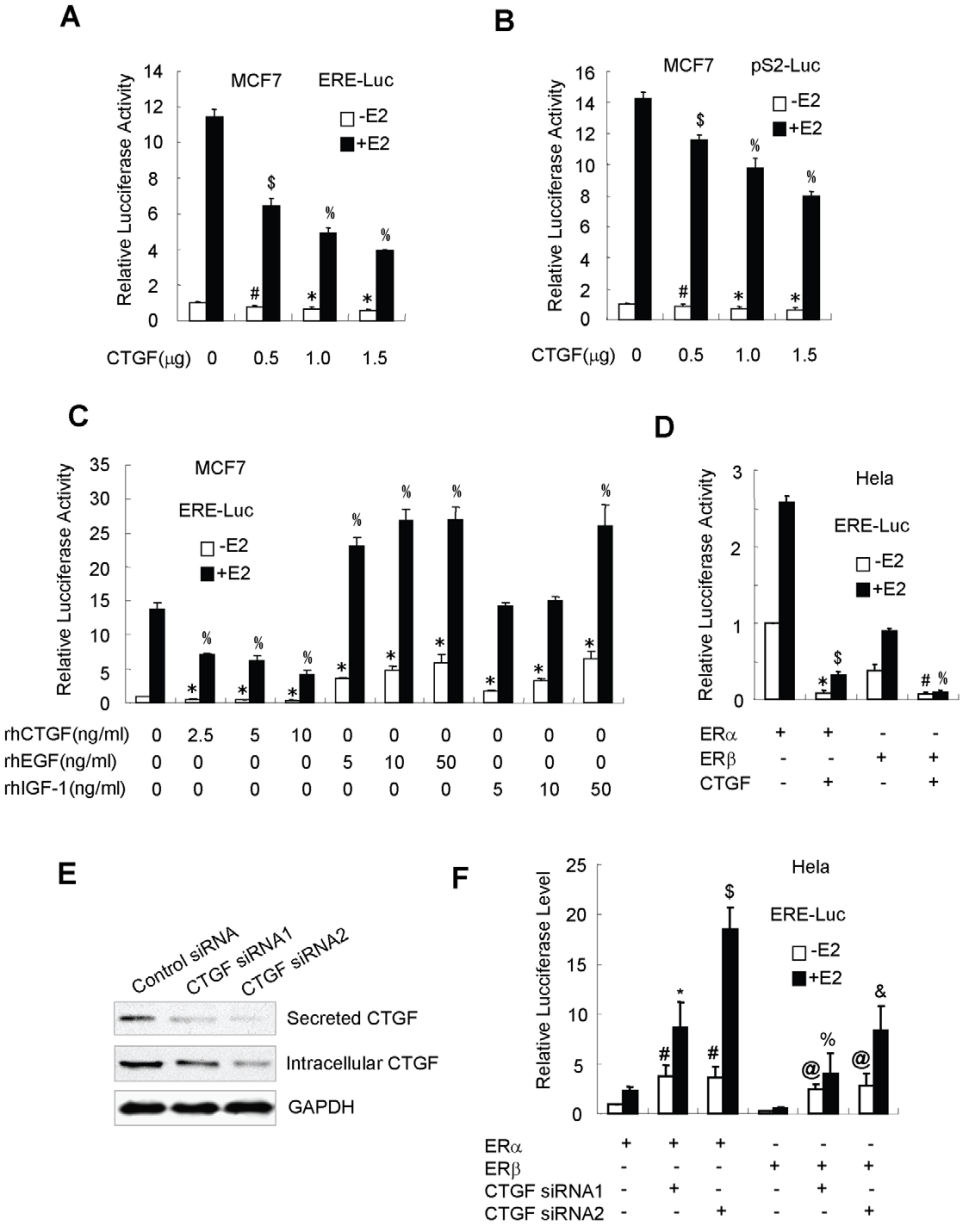
CTGF regulates estrogen-responsive reporter activity. (**A–C**) MCF7 cells were cotransfected with ERE-Luc (A and C) or pS2-Luc (B) reporter, and increasing amounts of plasmid expressing FLAG-tagged CTGF (A and B) or the indicated amounts of recombinant human (rh) CTGF, EGF or IGF-1 proteins (C). Cells were treated with or without 10 nm E2 for 24 h and analyzed for luciferase activity. Data shown are means ± SD of triplicates of one representative experiment and have been repeated three times with similar results. ^#^ *P*<0.05 versus empty vector without E2. *P<0.01 versus empty vector without E2. ^$^ *P*<0.05 versus empty vector with E2. ^%^ *P*<0.01 versus empty vector with E2. (D) Hela cells were co-transfected with ERE-Luc, FLAG-tagged CTGF, and ERα or ERβ as indicated. Cells were treated and analyzed as in (A–C). *P<0.01 versus ERα expression vector without E2. ^$^ *P*<0.01 versus ERα expression vector with E2. ^#^ *P*<0.01 versus ERβ expression vector without E2. ^%^ *P*<0.01 versus ERβ expression vector with E2. (E) Hela cells were transfected with expression vector for CTGF siRNA1, CTGF siRNA2 or scramble siRNA (control) plasmid. Cells were harvested and lysed, and conditioned medium was concentrated using a 10-kDa membrane. Both the concentrate and the cell lysate were used for immunoblotting of the expression of CTGF, and the whole cell lysate was used for immunoblotting of the expression of GAPDH. (F) Hela cells were cotransfected with ERE-Luc, ERα or ERβ, and CTGF siRNA1 or CTGF siRNA2, as indicated. Cells were treated and analyzed as in (A–C). ^#^ *P*<0.05 versus ERα expression vector without E2. *P<0.05 versus ERα expression vector with E2. ^$^ *P*<0.01 versus ERα expression vector with E2. ^@^ *P*<0.05 versus ERβ expression vector without E2. ^%^ *P*<0.05 versus ERβ expression vector with E2. ^&^ *P*<0.01 versus ERβ expression vector with E2.

Having firmly established that CTGF is an ERα- and ERβ-binding protein, we tested the effect of CTGF overexpression on the transcriptional activity of ERα and ERβ. ERα- and ERβ-positive MCF7 cells were cotransfected with the reporters, ERE-Luc (an artificial estrogen-responsive element-containing reporter) or pS2-Luc (a natural pS2 promoter-containing reporter), and increasing amounts of FLAG-tagged CTGF. As expected, E2 stimulated the endogenous ERα- and ERβ-mediated transcriptional activity (Fig. 4 A and B). Importantly, in both the presence and the absence of E2, overexpression of CTGF decreased both reporter activities in a dose-dependent manner. Similar results were observed in the ZR75-1 cell line, another human ERα-positive breast cancer cell line (data not shown, but see below). Moreover, recombinant human CTGF protein at similar levels to CTGF physiological concentrations in MCF7 cells also decreased the ERE-Luc activity, whereas recombinant EGF and IGF-1 proteins increased the ERE-Luc activity as previously reported [6], [7] (Fig. 4C).

To exactly determine the effect of CTGF overexpression on the transcriptional activity of ERα and ERβ, ERα- and ERβ-negative human Hela cervical cancer cells were cotransfected with the ERE-Luc reporter, ERα or ERβ, and FLAG-tagged CTGF. As shown in Fig. 4D, CTGF overexpression inhibited both ERα- and ERβ-dependent ERE-Luc reporter activities.

To test whether CTGF is a general repressor of nuclear receptor action, the effects of CTGF on the transcriptional activities of other nuclear receptors, such as androgen receptor (AR) and glucocorticoid receptor (GR), were investigated. MCF7 cells were cotransfected with FLAG-tagged CTGF and the ARE-Luc (androgen-responsive element-containing luciferase reporter) or pFC31-Luc (glucocorticoid-responsive element-containing luciferase reporter) reporter. As expected, R1881, a synthetic androgen, stimulated endogenous AR-mediated transcriptional activity (Fig. S2A), and dexamethasone (Dex), a synthetic gulcocorticoid, activated endogenous GR-mediated transcriptional activity (Fig. S2B). However, CTGF had no effect on transactivation function of both AR and GR, suggesting that CTGF specifically regulates ER transcriptional activity.

### Knockdown of endogenous CTGF increases the transcriptional activity of ERα and ERβ

To investigate the role of endogenous CTGF in regulation of ERα- and ERβ-mediated transcriptional activity, Hela cells, which expressed high level of CTGF, were transfected with vector-based CTGF siRNAs or universal scramble siRNA (control). As shown in Fig. 4E, CTGF siRNA1 and CTGF siRNA2 effectively repressed the expression of CTGF to varying degrees, whereas universal scramble siRNA had no effect. In agreement with the inhibitory effects of both CTGF siRNAs, suppression of the normal expression of CTGF in Hela cells by the specific CTGF siRNAs significantly increased the ERα- or ERβ-mediated ERE-Luc reporter activity (Fig. 4F). These results further suggest that CTGF decreases the transcriptional activity of ERα and ERβ.

### CTGF decreases the expression of endogenous estrogen-responsive genes

**Figure 5:**
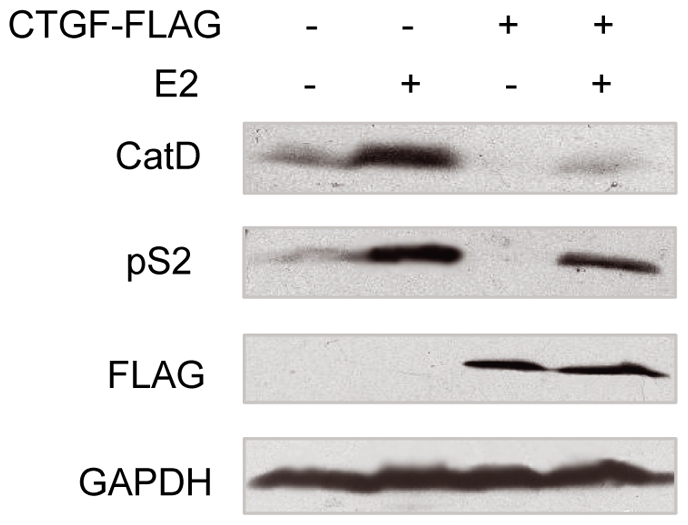
CTGF reduces estrogen-responsive protein expression. MCF7 cells stably transfected with FLAG-tagged CTGF were treated with E2 or without E2. Conditioned media were blotted with antibodies to cathepsin D (CatD), pS2 and FLAG, and whole cell lysates were blotted with anti-GAPDH.

To corroborate the results of the luciferase reporter assays, the effect of CTGF on the expression of endogenous estrogen-responsive genes was examined. The E2-deprived MCF-7 cells stably expressing either the empty vector or FLAG-tagged CTGF were treated with 10 nM E2 for 20 h. As expected, E2 increased the expression of two well-studied estrogen-responsive genes [3], pS2 and cathepsin D, in the empty vector-transfected cells (Fig. 5). Importantly, the transfection of CTGF decreased the expression of pS2 and cathepsin D both in the absence and in the presence of E2. These data suggest that CTGF represses the expression of endogenous ERα-responsive genes.

### Secreted CTGF is critical for repression of ER transcriptional activity

**Figure 6:**
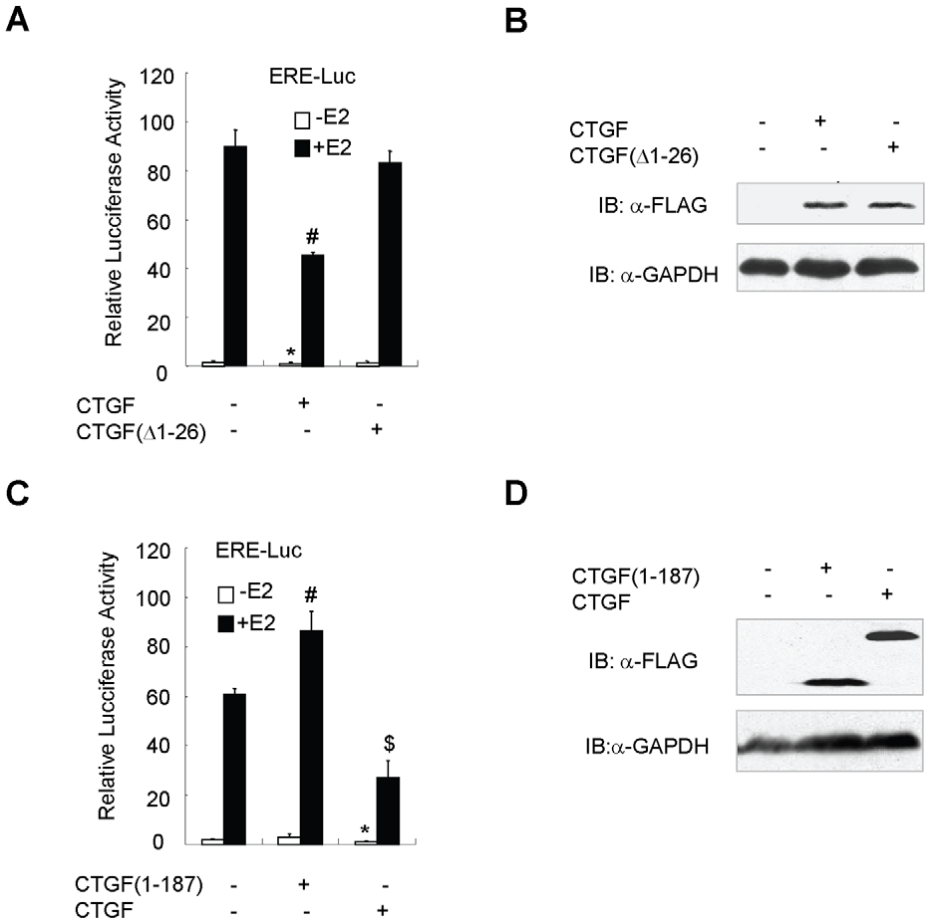
Effects of CTGF deletion mutants on estrogen-responsive reporter activity. (A) Luciferase reporter assay with the CTGF deletion mutant without the signal peptide. ZR75-1 cells were cotransfected with ERE-Luc and FLAG-tagged CTGF or CTGF(Δ1-26) as indicated. Cells were treated and analyzed as in Fig. 4A. *P<0.01 versus empty vector without E2. ^#^ *P*<0.01 versus empty vector with E2. (B) Western blotting showing expression levels of FLAG-tagged CTGF and CTGF(Δ1-26) with antibody against FLAG or GAPDH. Cells were transfected in the presence of E2 as in (A). (C) Luciferase reporter assay with the CTGF deletion mutant lacking ER-binding site. Cells were cotransfected with ERE-Luc and FLAG-tagged CTGF or CTGF(1–187) as indicated. *P<0.01 versus empty vector without E2. ^#^ *P*<0.05 versus empty vector with E2. ^$^ *P*<0.01 versus empty vector with E2. (D) Western blotting showing expression levels of FLAG-tagged CTGF and CTGF(1–187) with antibody against FLAG or GAPDH. Cells were transfected in the presence of E2 as in (C). Conditioned medium was used for Western blotting analysis of the expression of FLAG-tagged CTGF and CTGF(1–187), and the whole cell lysate was used for Western blotting analysis of the expression of GAPDH.

As CTGF is a secreted protein, we used the constructs for wild-type CTGF and CTGF without the signal peptide to test if CTGF regulates ER transcriptional activity through autocrine and/or paracrine mechanisms. ERα-positive ZR75-1 cells were transfected with the constructs for FLAG-tagged CTGF or CTGF without the signal peptide [CTGF(Δ1-26)]. Unlike FLAG-tagged CTGF, CTGF(Δ1-26) could not be secreted into medium (data not shown). ZR75-1 cells were then cotransfected with the ERE-Luc reporter and FLAG-tagged CTGF or CTGF(Δ1-26). As shown in Fig. 6A, CTGF markedly inhibited the reporter activity, whereas CTGF(Δ1-26) abrogated the ability of CTGF to repress the activity. It should be noted that FLAG-tagged CTGF and CTGF(Δ1-26) were expressed at comparable levels (Fig. 6B). These data suggest that secreted CTGF, but not cytoplasmic CTGF, is responsible for repression of ER transcriptional activity.

### The interaction of CTGF and ER is required for repression of estrogen-responsive transcription

To examine whether the interaction between CTGF and ER is necessary for the regulation of estrogen-responsive transcription, the CTGF mutant [CTGF(1–187)] which failed to interact with ER was used. MCF7 cells were cotransfected with the ERE-Luc reporter and FLAG-tagged full-length CTGF or CTGF(1–187). As shown in Fig. 6C, the CTGF(1–187) lacking the ER-binding site completely abolished the CTGF repression of the reporter activity. In contrast, the CTGF(1–187) slightly increased the reporter activity. Notably, both FLAG-tagged CTGF and CTGF(1–187) could be secreted into medium and were expressed at comparable levels (Fig. 6D). These data suggest that the interaction between CTGF and ER is required for repression of estrogen-responsive transcription by CTGF.

### CTGF did not affect ERα binding to ERE sequence

**Figure 7:**
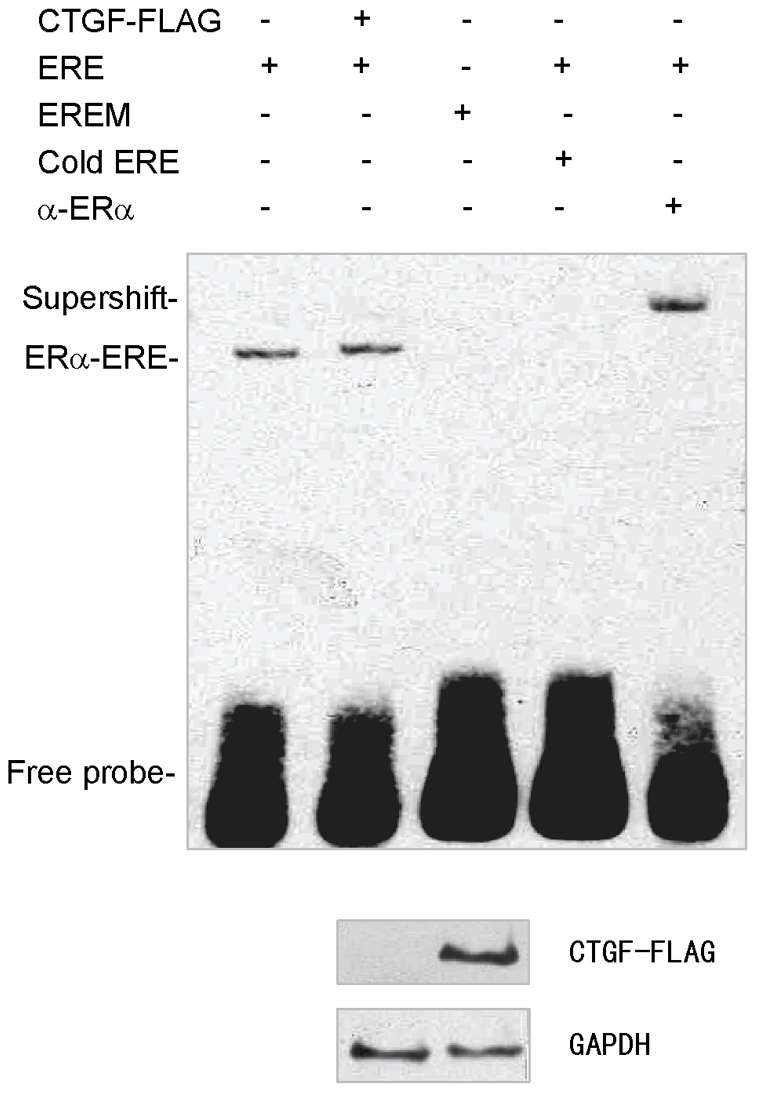
Effect of CTGF on ERα binding to ERE sequence. EMSA was performed using biotin-labeled ERE probe and nuclear proteins extracted from ZR75-1 cells transfected with empty vector or FLAG-tagged CTGF in the presence of 10 nM E2. For competition experiments, a 100-fold molar excess of unlabeled ERE was incubated with the labeled probe. The biotin-labeled mutant ERE probe (EREM) was used as a negative control. Supershifts were performed using specific anti-ERα antibody. The representative immunoblot with anti-FLAG shows the expression level of intracellular FLAG-tagged CTGF (lower panel).

To investigate molecular mechanism by which CTGF modulates ER transcriptional activity, the effect of CTGF on ER**α** binding to ERE sequence was determined by EMSA. As expected, the biotin-labeled ERE, but not mutant ERE (EREM), bound to proteins from ER-positive ZR75-1 nuclear extracts in the presence of E2 (Fig. 7). The binding was specifically inhibited by a 100-fold molar excess of a cold ERE oligonucleotide. The addition of human anti-ERα antibody to the reaction caused a supershift, indicating that ERα protein from ZR75-1 nuclear extracts specifically binds to ERE sequence. However, overexpression of CTGF did not affect the binding of ERα to ERE (Fig. 7), suggesting the involvement of other mechanism(s) in CTGF modulation of ER transcriptional activity.

### CTGF inhibits ERα expression

**Figure 8:**
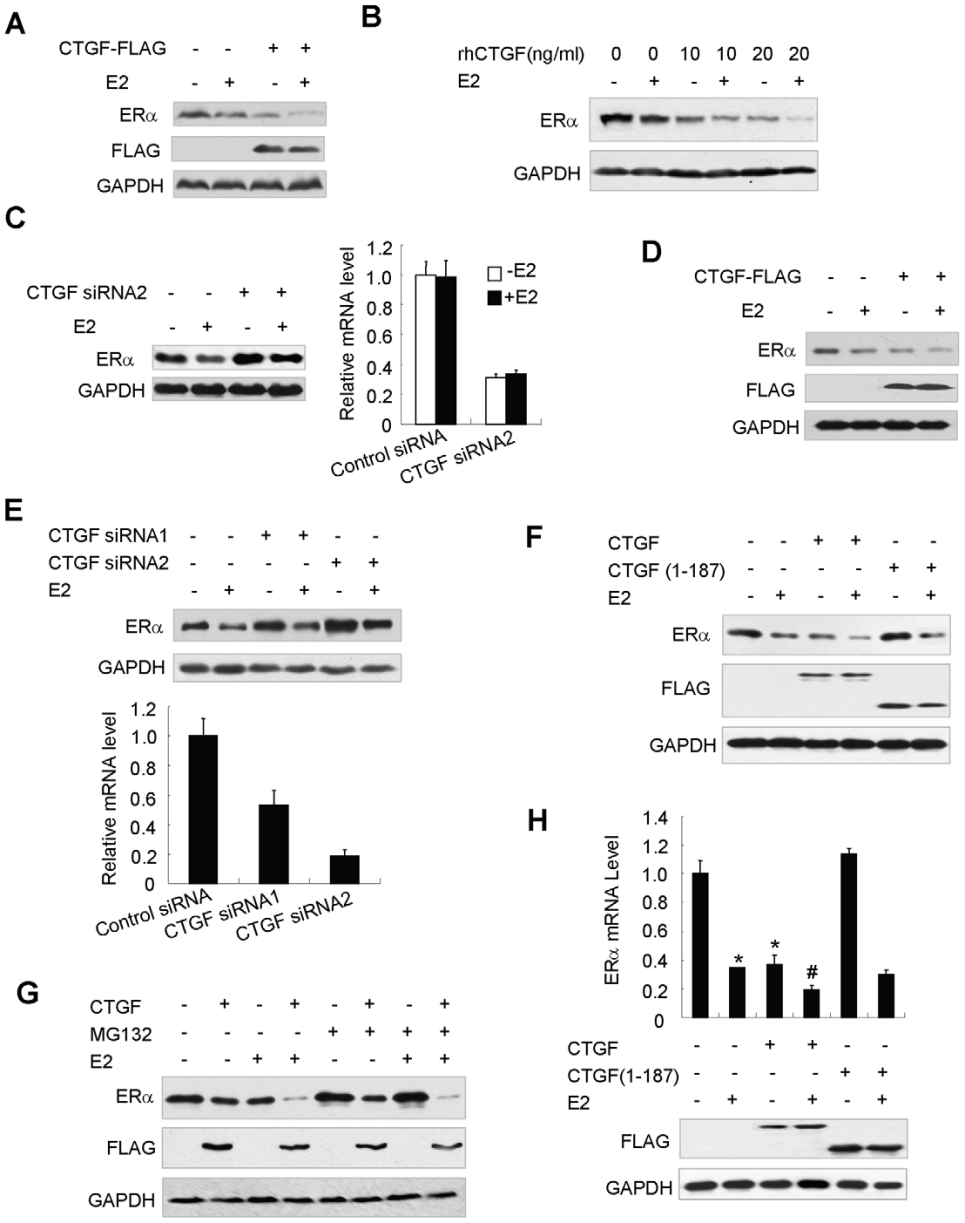
CTGF inhibits ERα expression. (A) MCF7 cells stably transfected with FLAG-tagged CTGF, which constitutively expressed FLAG-CTGF, or MCF7 cells stably transfected with empty vector were treated with 10 nm E2 for 24 h. Whole cell lysates were blotted with the indicated antibodies. (B) MCF7 cells were treated with the indicated amounts of recombinant human CTGF (rhCTGF) and analyzed as in (A). (C) MCF7 cells transfected with CTGF siRNA2 or control siRNA were treated and analyzed as in (A). Knockdown effect of CTGF siRNA2 on the endogenous CTGF mRNA level was determined by real-time PCR with CTGF and β-actin primers (right panel). (D) ZR75-1 cells transiently transfected with FLAG-tagged CTGF or empty vector were treated and analyzed as in (A). (E) ZR75-1 cells transfected with CTGF siRNA1 or CTGF siRNA2 were treated and analyzed as in (A). Knockdown effect of CTGF siRNA1 or CTGF siRNA2 on the endogenous CTGF mRNA levels was determined as in (C) (lower panel). (F) MCF7 cells were transfected with FLAG-tagged CTGF or CTGF(1–187) and treated with 10 nm E2. Cell lysates were analyzed by immunoblot with the indicated antibodies. (G) MCF7 cells stably transfected with FLAG-tagged CTGF or empty vector were pretreated with 10 µM MG132 for 1 h to block proteasome activity. Cells were then treated with 10 nM E2 for 24 h. Cell lysates were analyzed as in (D). (H) MCF7 cells transfected with FLAG-tagged CTGF or CTGF(1–187) were used for real-time RT-PCR with ERα and GAPDH primers (upper panel). Cell lysates were examined by immunoblot with the indicated antibodies (lower panel). Data shown are means ± SD of triplicates of one representative experiment and have been repeated three times with similar results. *P<0.01 versus empty vector without E2. ^#^ *P*<0.01 versus empty vector with E2.

To further investigate the mechanisms by which CTGF represses ER transcriptional activity, we determined the effect of CTGF on ERα expression by immunoblotting. As expected [18], E2 decreased ERα protein levels in MCF7 or ZR75-1 cells (Fig. 8 A–E). Importantly, Both FLAG-tagged CTGF and recombinant human CTGF inhibited ERα protein expression both in the presence and in the absence of estrogen, and recombinant human CTGF inhibited ERα protein expression in a dose-dependent manner (Fig. 8 A, B and D). In contrast, knockdown of endogenous CTGF in MCF7 or ZR75-1 cells increased ERα protein levels (Fig. 8 C and E). Although FLAG-tagged full-length CTGF repressed the expression of ERα protein, the CTGF(1–187) mutant that did not decrease ERα transcriptional activity also did not change ERα protein levels in MCF7 cells (Fig. 8F). Reduction of ERα protein levels by CTGF is not mediated through proteosome-dependent protein degradation because MG132, a proteosome inhibitor, had no effect on CTGF-mediated repression of ERα protein expression (Fig. 8G). As a control, MG132 blocked E2-induced dowregulation of ERα.

Next, we investigated the effect of CTGF on ERα mRNA expression. As previously reported [19], E2 decreased ERα mRNA levels in MCF7 cells (Fig. 8H and Fig. S3). Intriguingly, CTGF reduced ERα mRNA expression both in the presence and in the absence of estrogen, whereas the CTGF(1–187) mutant that did not repress ERα transcriptional activity also did not alter ERα mRNA levels in MCF7 cells. Taken together, these results suggest that CTGF may regulate ERα expression at the mRNA level and that CTGF inhibits ERα transcriptional activity at least in part through decreased ERα expression.

## Discussion

Estrogen plays a critical role in regulating the growth, differentiation, and function of tissues of the reproductive system, including the mammary glands, uterus, vagina, and ovaries in females, and the testis, epididymis, and prostate in males. Estrogen exerts its biological function through ERs acting via classical genomic events in the nucleus and by non-genomic actions at the plasma membrane [20]. Although the exact nature of membrane ERs remains to be characterized, increasing evidence indicates that the non-genomic actions of estrogen involve the classical ERs located at the plasma membrane [21]–[23]. Membrane ERα mediates non-genomic estrogen actions by forming a complex with many signaling molecules, such as the regulatory subunit of phosphatidylinositol-3-OH kinase (PI3K), Shc, IGF-1R, SRC, and caveolin-1, leading to indirect activation of ER transcriptional activity.

ER genomic activity is also enhanced by various growth factor signaling pathways, such as EGF, IGF-1 and transforming growth factor α (TGFα) [6], [7]. These peptide growth factors, which are extracellular ligands, induce transcriptional activation of a concensus estrogen response element (ERE) in an ER-dependent manner in various cell types, including breast cancer and ovarian cancer cell lines. The growth factors and estrogen synergistically enhance ER transactivation function although there is no synergism between the different classes of growth factors, such as TGFα and IGF-1. Unlike EGF and IGF-1, which increases ER transcriptional activity, CTGF, another growth factor, represses ER transcriptional activity both in the presence and in the absence of estrogen. To the best of our knowledge, CTGF is the first growth factor to inhibit ER transcriptional activation.

IGF-1R has been shown to physically interact with ER after estrogen treatment [24]. Since IGF-1 is a ligand for IGF-1R, it is possible that IGF-1 increases ER transcriptional activity through its interaction with IGF-1R. Whether IGF-1, IGF-1R and ER form a complex remains to be elucidated. In this study, we present evidence of physical and functional interactions between CTGF and ER. The physical interaction has been validated by a number of in vitro and in vivo experiments, including yeast two-hybrid, in vitro GST pull-down, in vivo co-immunoprecipitation, ELISA, and immunofluorescence. Importantly, CTGF directly associates with ER. Moreover, we can demonstrate that CTGF functionally inhibits ER transcriptional activity, suggesting that CTGF is a novel repressor of ER signaling. Secreted CTGF, but not cytoplasmic CTGF, is critical for repression of ER transcriptional activity. Secreted wild-type CTGF that interacts with ER can repress ER transcriptional activity, whereas the secreted CTGF mutant that fails to interact with ER also fails to inhibit ER transcriptional activity. We believe that CTGF is the first molecule of this class to be identified, but most likely there will be more to come. This notion may be supported by the fact that membrane ER activates multiple intracellular signaling pathways and peptide growth factors cross-talk with ER signaling [4], [5], [25].

The ERα-interacting region in CTGF is mapped to the thrombospondin type I repeat (TSP-1), which is thought to be a cell attachment motif [8]–[10]. CTGF interacts with ERα DNA-binding domain (DBD). ERα has been reported to interact with a number of co-factors, including co-activators and co-repressors [26], [27]. Most of ERα co-factors interact with ERα ligand-binding domain (LBD), whereas very few ERα co-factors interact with the DBD. The ERα DBD-interacting proteins include the co-activator X box-binding protein 1 (XBP-1) [13], which regulates ERα signaling both in the absence and in the presence of estrogen, and the co-repressors template-activating factor Iβ (TAF-Iβ) [28], pp32 [29], and zinc finger protein 366 (ZNF366) [30]. Like these co-repressors, CTGF inhibits ERα transcriptional activity. Since the DBD domain of ERα has 96% homology with that of ERβ, it is not surprising that both ERα and ERβ bind to CTGF.

It has been reported that integrins ανβ3, αIIbβ3, αMβ2 and α5β1, and low density lipoprotein receptor-related protein/α2-macroglobulin receptor (LRP) are cell surface receptors of CTGF [31], [32]. Through binding to these cell surface proteins, CTGF exerts a range of diverse biological functions, including proliferation, differentiation, apoptosis, cell adhesion, migration, and angiogenesis. CTGF interacts with the Wnt receptor complex, including the Wnt receptor Frizzled 8 and the Wnt co-receptor LRP6, and inhibits Wnt signaling, which is pivotal to gene expression, cell adhesion, tissue development and oncogenesis [33]. The C-terminal (CT) domain of CTGF, which is not necessary for binding ER, is required for binding LRP6 and complete inhibition of Wnt signaling by CTGF. There are at least 19 Wnt family members that signal through complexes comprising the Frizzled family of cell surface receptors together with LRP family members, which serve as co-receptors. Several lines of evidence demonstrated cross-talk between Wnt and estrogen signaling pathways [34]–[37]. Both Cyclin D1 and c-Myc, important regulators of cell proliferation, are known targets for both Wnt and estrogen pathways [35]. Estrogen rapidly increases the expression of Wnt-4 and Wnt-5a of the Wnt family and frizzled 2 of the Wnt receptor in the mouse uterus in an ER-independent manner, and the estrogen-dependent control of Wnt signaling then regulates late uterine growth response that is ER dependent [36]. Interestingly, ERα expression was restored at both mRNA and protein level after treatment of ERα-negative breast cancer cells with Wnt-5a [37]. Whether CTGF integrates estrogen and Wnt signaling remains to be investigated.

Several lines of evidence support important roles for CTGF in cancer development and progression. Over-expression of CTGF is found in prostate cancers [38], gliomas [39] and esophageal squamous cell carcinoma [40], and promotes tumor cell proliferation as well as tumorigenecity. In sharp contrast, CTGF expression is down-regulated in lung [41], colon [42] and ovarian [43] cancers. Over-expression of CTGF inhibits the growth of ovarian cancer cells as well as invasion and metastasis of lung and colon cancer cells in vitro and in vivo. Taken together, these data suggest that the role of CTGF in cancer development and progression is dependent on cancer or cell types. Contradictory results have been reported on the role of CTGF in breast cancer. Jiang et al. showed that, in addition to lower levels of CTGF in breast cancer tissues (122 cases) compared with normal tissues (32 cases), markedly reduced levels of CTGF in breast cancer patients are associated with poor prognosis, metastasis, local recurrence and mortality [44], whereas Xie et al. demonstrated that, compared with normal breast (7 cases), elevated levels of CTGF in primary breast cancer (44 cases) was observed [45]. Several studies about the effects of CTGF on breast cancer cell growth, migration and metastasis also produced conflicting results. The study by Hishikawa showed that forced expression of CTGF in MCF7 breast cancer cells stimulates apoptosis [46]. However, Chen et al. reported that CTGF increases the motility of breast cancer cells [31]. Another study by Kang et al. indicated that over-expression of CTGF alone in human breast cancer MDA-MB-231 cells did not cause a significant increase in bone metastasis formation, whereas over-expression of CTGF together with interleukin-11 (IL-11) and osteopontin (OPN) showed a dramatic increase both in the rate and in the incidence of bone metastases [47]. A recent study demonstrated that over-expression of the genome organizer protein SATB1, which is over-expressed in aggressive breast tumors, stimulates CTGF expression [48]. TGF-β, a cytokine that inhibits growth of normal epithelia and early stage tumors but stimulates invasion and metastasis of aggressive tumors, also increases CTGF expression. It is unclear whether CTGF has dual effects like TGF-β. Therefore, it will be interesting to determine the biological significance of CTGF repression of ER transcriptional activity in cancer development and progression.

The fact that CTGF can inhibit ERα expression in breast cancer cell lines suggests that ERα-negative breast cancers might have high levels of CTGF, whereas ERα-positive breast cancers might have low levels. Jiang et al. showed that, although there was no significant correlation between ERα and CTGF when breast tumors were analyzed as an entire cohort, ERα was inversely correlated with CTGF in tumor-node-metastasis (TNM) 3 breast tumors [44]. Generally speaking, the TNM 3 breast cancer group is more aggressive than TNM groups 1 and 2. It has been reported that ERα-positive breast cancers are often responsive to anti-estrogen therapy and generally have a better prognosis, while ERα-negative breast cancers are more aggressive and unresponsive to anti-estrogens [1]. Our present findings raise the possibility that, at least in a subset of breast cancer patients, CTGF might contribute to the process of breast cancer progression by allowing the development of ERα-negative phenotypes through reduction of ERα expression and repression of ER transcriptional activity, resulting in enhanced aggressiveness of breast cancer cells. Large clinical samples are needed to exactly elucidate the correlation of CTGF with ERα in breast cancer.

## Supporting Information

Figure S1 **Characterization of anti-CTGF antibody for Immunofluorescence.** (A) Anti-CTGF was pre-incubated with His control or approximately 10 µg of His-tagged CTGF protein (His-CTGF) for 1 h and then used for immunofluorescence analysis of MCF7 cells stably transfected with FLAG-tagged CTGF. The nuclei were stained with DAPI. The CTGF expression was visualized by fluorescence microscopy (Left panel). Original magnification, ×200. Scale bar, 50 µm. SDS-PAGE analysis of the purified His-CTGF protein is shown in the right panel. (B) MCF7 cells or MCF7 cells stably transfected with FLAG-tagged CTGF were stained with the anti-CTGF antibody and analyzed as in (A).
(TIF)

Figure S2 **Effect of CTGF on the transcriptional activities of AR and GR.** MCF7 cells were cotransfected with FLAG-tagged CTGF and the ARE-Luc (A) or pFC31-Luc (B) reporter. Cells were treated with or without 0.1 nm R1881 or 0.1 µM Dex for 24 h and analyzed for luciferase activity. Data shown are means ± SD of triplicates of one representative experiment and have been repeated three times with similar results.
(TIF)

Figure S3 **CTGF suppresses ERα mRNA expression.** MCF7 cells were transfected with FLAG-tagged CTGF or CTGF(1–187) as in Figure 8 and were used for real-time RT-PCR with ERα and β-actin primers. Data shown are means ± SD of triplicates of one representative experiment and have been repeated three times with similar results. *P<0.01 versus empty vector without E2. ^#^P<0.01 versus empty vector with E2.
(TIF)
