# Supplementary figures and images for: Suppression of Estrogen Receptor Transcriptional Activity by Connective Tissue Growth Factor
Source: PLoS One. 2011 May 24;6(5):e20028. doi: 10.1371/journal.pone.0020028 (PMC3101213; doi:10.1371/journal.pone.0020028)

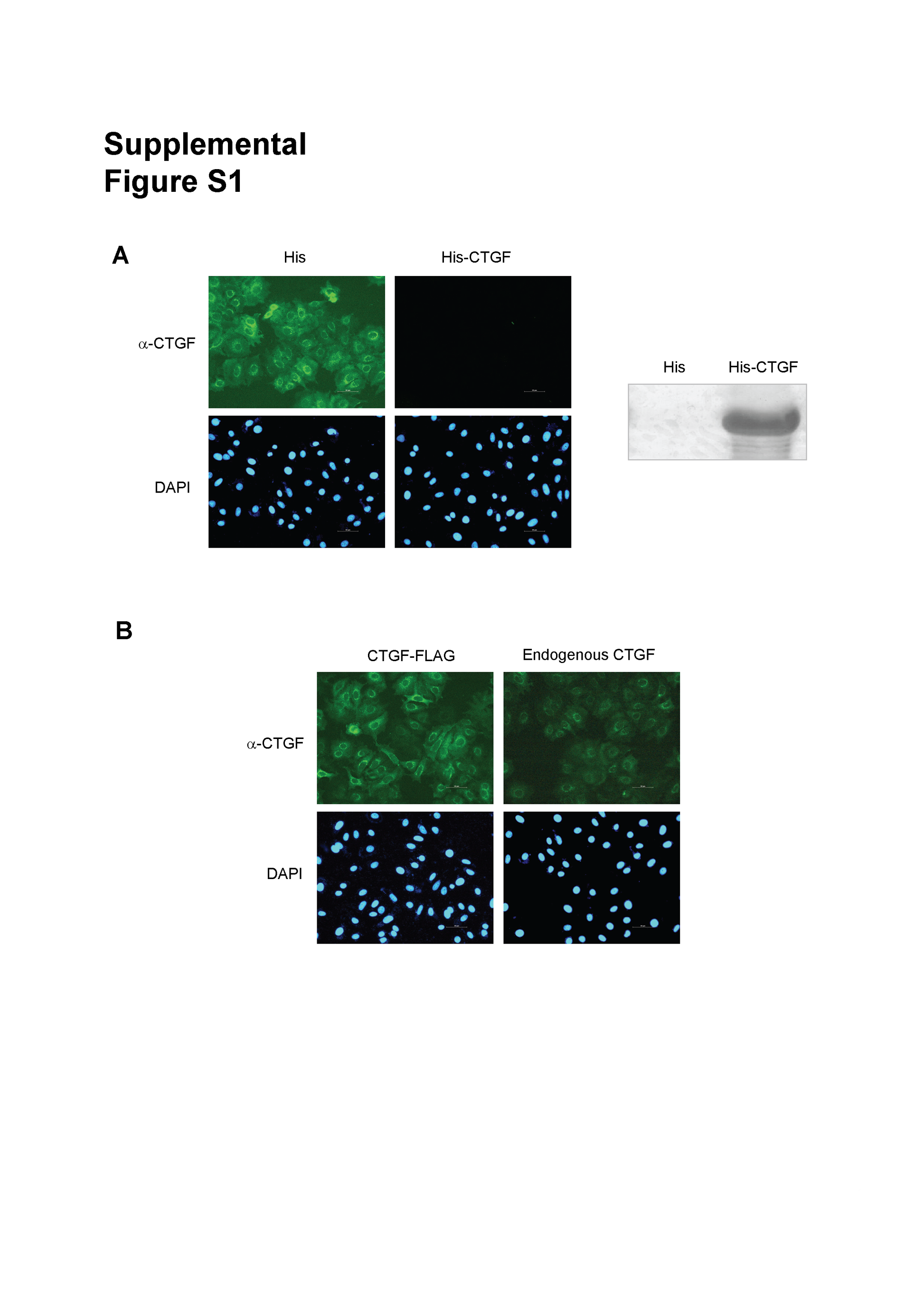

Supplement: Figure S1 — Characterization of anti-CTGF antibody for Immunofluorescence. (A) Anti-CTGF was pre-incubated with His control or approximately 10 µg of His-tagged CTGF protein (His-CTGF) for 1 h and then used for immunofluorescence analysis of MCF7 cells stably transfected with FLAG-tagged CTGF. The nuclei were stained with DAPI. The CTGF expression was visualized by fluorescence microscopy (Left panel). Original magnification, ×200. Scale bar, 50 µm. SDS-PAGE analysis of the purified His-CTGF protein is shown in the right panel. (B) MCF7 cells or MCF7 cells stably transfected with FLAG-tagged CTGF were stained with the anti-CTGF antibody and analyzed as in (A). (TIF) [file pone.0020028.s001.tif]

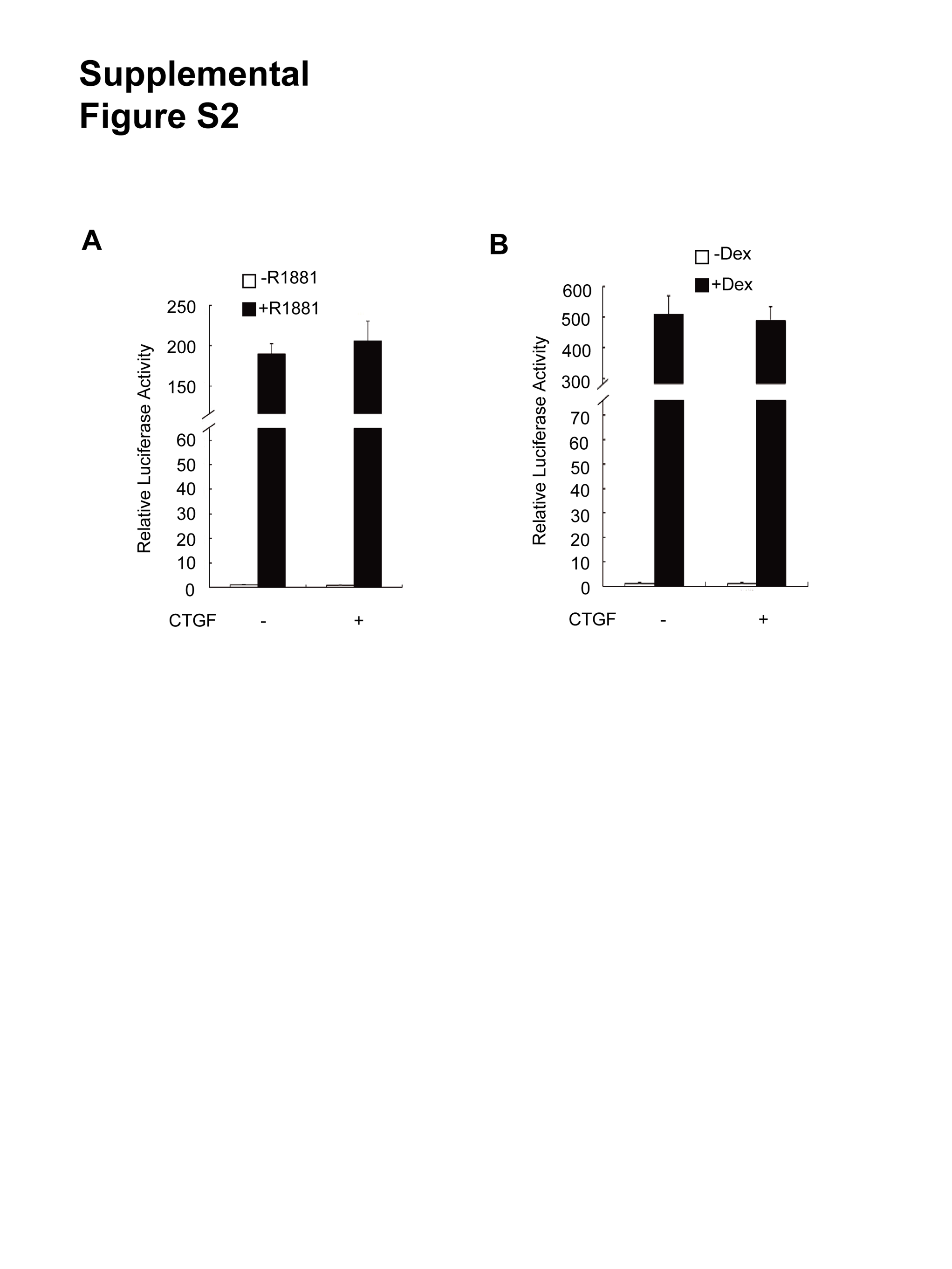

Supplement: Figure S2 — Effect of CTGF on the transcriptional activities of AR and GR. MCF7 cells were cotransfected with FLAG-tagged CTGF and the ARE-Luc (A) or pFC31-Luc (B) reporter. Cells were treated with or without 0.1 nm R1881 or 0.1 µM Dex for 24 h and analyzed for luciferase activity. Data shown are means ± SD of triplicates of one representative experiment and have been repeated three times with similar results. (TIF) [file pone.0020028.s002.tif]

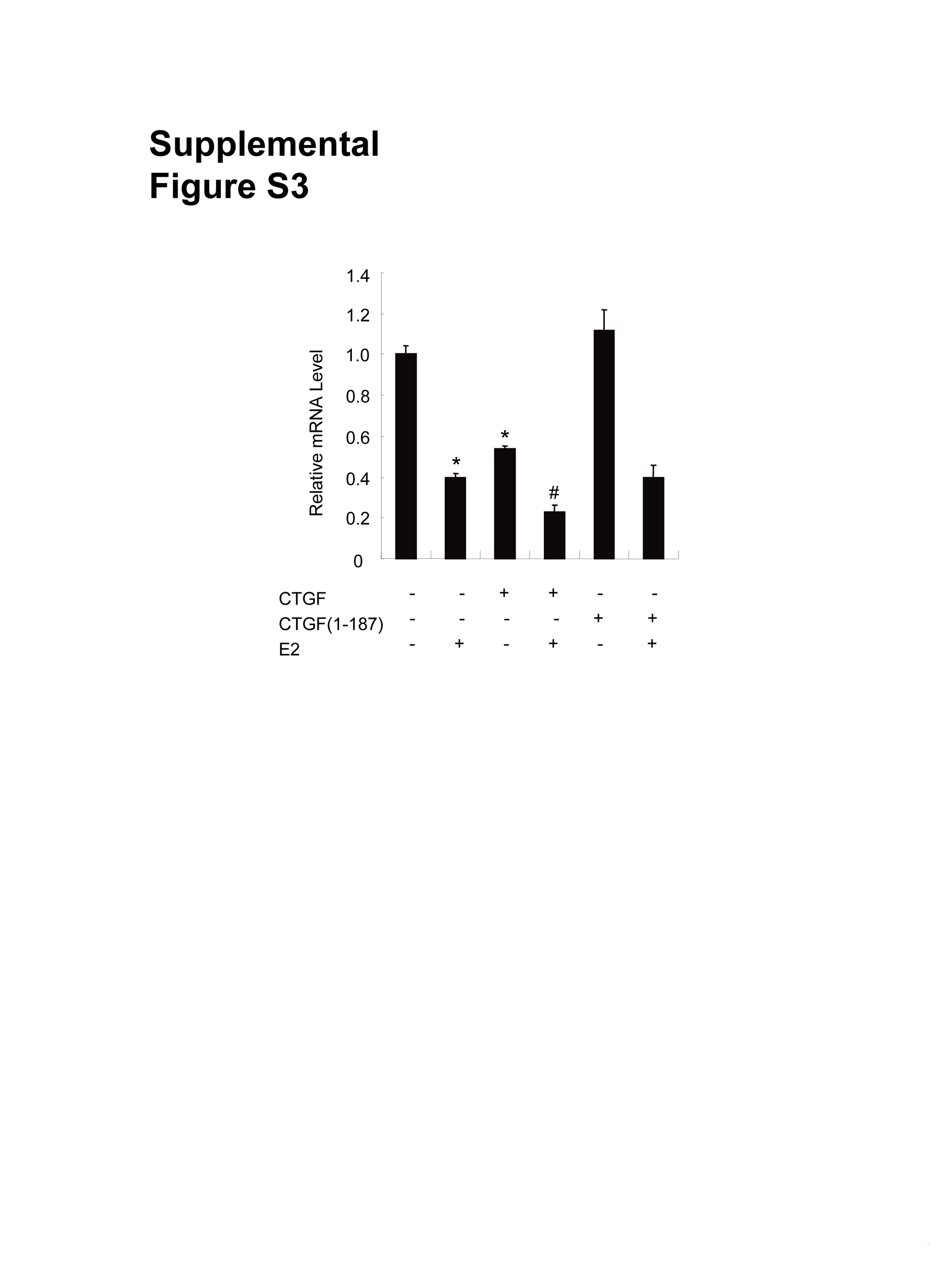

Supplement: Figure S3 — CTGF suppresses ERα mRNA expression. MCF7 cells were transfected with FLAG-tagged CTGF or CTGF(1–187) as in Figure 8 and were used for real-time RT-PCR with ERα and β-actin primers. Data shown are means ± SD of triplicates of one representative experiment and have been repeated three times with similar results. *P<0.01 versus empty vector without E2. #P<0.01 versus empty vector with E2. (TIF) [file pone.0020028.s003.tif]
